# Oral hygiene indices and chronic kidney disease in children: Systematic review and *meta*-analysis of case-control studies

**DOI:** 10.1016/j.sdentj.2024.11.009

**Published:** 2024-12-09

**Authors:** Narjes Amrollahi, Mohammad javad Tarrahi, Zahra Abbasi

**Affiliations:** a Assistant Professor. Dental Research Center, Department of Pediatric Dentistry, Dental Research Institute, School of Dentistry, Isfahan University of Medical Sciences, Isfahan, Iran; b Department of Epidemiology and Biostatistics, School of Health, Isfahan University of Medical Sciences, Isfahan, Iran; c Dentist, Dental Students’ Research Committee, School of Dentistry, Isfahan University of Medical Sciences, Isfahan, Iran

**Keywords:** Child, Chronic kidney insufficiency, Oral hygiene

## Abstract

**Background:**

Chronic kidney disease (CKD) is a disorder that causes numerous problems for children and affects many organs, as oral hard and soft tissues. The purpose of this study was to summarize the relationship between oral and dental health status and CKD in children.

**Materials and Methods:**

A systematic search of texts from 2000 to 2023 was conducted to gather all case control studies published in the English language related to the subject of this study in PubMed, Web of Science, Scopus and Cochrane electronic databases. The title, abstract and full text of the articles were examined according to inclusion criteria until relevant studies were selected. This process was carried out independently by two researchers. The quality of the selected studies was assessed by the National Institutes of Health (NIH) checklist.

**Results:**

A total of 768 articles were identified in electronic databases during the search process. Articles inconsistent with inclusion criteria and duplicate articles were removed and 8 studies were selected for *meta*-analysis. The results found no statistically significant difference in mean of decayed, missing and filled teeth in permanent (DMFT index) and primary teeth (dmft index) in CKD and healthy children with a mean difference of −0.433 (95 % CI: −1.689 to 0.823; p-value = 0.500) and-0.095 (95 % CI: −2.240 to 2.051; p-value = 0.931) respectively. However, CKD had a significant effect on the developmental defects of enamel (DDE) index in children with an effect size of 4.916 (95 % CI: 1.752 to 13.799; p-value = 0.002).

**Conclusion:**

The presence of CKD can increase the incidence of DDE by 4.9 times, but it has no significant effect on the prevalence of dental caries in primary and permanent teeth.

## 1 Introduction

Chronic kidney disease (CKD) is a disorder that causes many problems for children. As a complex disease, its treatment is very expensive. Kidney disease affects many organs, as oral hard and soft tissues. It also decreases the salivary flow and increases the symptoms and discomforts such as dry mouth and burning sensation in the mouth (BMS) (Vesterinen et al., 2012).

Other oral manifestations including an ammonia-like odor, taste disturbance, stomatitis, and secondary gingival enlargement after drug treatment are seen in these children. Decreased salivary flow also makes patients prone to oral and dental problems such as dental caries, periodontal disease and Candida infections (Nylund et al., 2018). Additionally, developmental defects of enamel (DDE) may be seen due to disturbances in calcium and phosphate metabolism. In these patients, oral manifestations such as gingivitis, excessive gingival growth and enamel hypoplasia may be seen more frequently (Davidovich et al., 2005).

CKD is one of the rapidly increasing chronic diseases. Nearly 500 million people suffer from CKD, with the majority (80 %) living in developing countries. The prevalence of CKD even in developed countries is reported to be 8.6 % in men and 9.6 % in women. Although children represent only a small population of all patients with CKD, they have special challenges because of the many extra renal manifestations of CKD that complicate the management (Sezer et al., 2023). Due to the increasing prevalence of this disease, it is crucial to pay attention to oral and dental hygiene for preventive planning in children suffering from CKD.

Numerous studies have been conducted to evaluate oral and dental health, including dental caries, gingival health, DDE and oral hygiene in children with CKD. There are some controversies in the results of these studies. In some studies, the prevalence of dental caries in children with CKD did not differ from healthy children (Baygin, 2017, Szulimowska et al., 2023). In other studies (Silva et al., 2019, Andaloro et al., 2018), lower prevalence of dental caries were reported in CKD children, which could be due to the presence of urea in the saliva. Urea has antibacterial and acid neutralizing capacity leading to lower prevalence of dental caries (Velan and Sheller, 2021). The prevalence of DDE was assessed in several studies. (Caliento et al., 2018, Silva et al., 2019, Sezer et al., 2023, Baygin, 2017). Also, studies on oral hygiene and gingival health in children with CKD have reported contradictory results. (Andaloro et al., 2018, Szulimowska et al., 2023, Davidovich et al., 2005, Caliento et al., 2018, Maciejczyk et al., 2019) The purpose of this study was to summarize the relationship between oral and dental health status and CKD in children.

## 2 Materials and methods

This systematic review was done base on the guidelines of Preferred Reporting Items for Systematic Reviews and Meta-Analysis (PRISMA statement) and was approved by the ethics committee of Isfahan University of Medical Sciences, Isfahan, Iran with the ID number of IR.MUI.RESEARCH.REC.1402.177.

### 2.1 Inclusion and exclusion criteria

Case-control studies that were conducted on the oral and dental health status of individuals between 3 to 18 years old with CKD were selected to include in the study. Other types of articles were not considered. Additionally, only articles written in English with both their abstracts and full texts available were included.

The exclusion criteria included the following items: cross sectional and case report articles, review studies, systematic reviews, letters, personal statements, book chapters, abstracts of conference articles, guidelines, pilot studies, articles in which children had other systemic or developmental diseases such as diabetes, history of transplantation, history of dialysis and hemodialysis. Studies written in languages other than English were also excluded.

### 2.2 Search strategy

A systematic search of texts from 2000 to 2023 was conducted to obtain all articles published in the English language related to the subject of this study in PubMed, Web of Science, Scopus and Cochrane electronic databases. The studies focused on children (P, population) with CKD (E, Exposure) compared to healthy children (C, comparison) assessing dental and oral hygiene indices as the outcome (O). The search strategy was created based on medical subject heading (MeSH) or non-MeSH key words and was as follows:

1. “Kidney Failure, Chronic”[Mesh] OR “Renal Insufficiency, Chronic”[Mesh] OR chronic renal disease OR “Kidney Diseases”[Mesh]
2. “Child”[Mesh] OR “Pediatric Dentistry”[Mesh] OR pediat OR “Dental Care for Children”[Mesh]
3. “Oral Hygiene”[Mesh] OR dental hygiene OR “Dental Caries”[Mesh] OR caries OR tooth decay OR DMFT OR tooth decay OR developmental enamel defect index OR “Dental Plaque Index”[Mesh] OR “Gingival Diseases”[Mesh] OR gingival index OR “Periodontal Diseases”[Mesh] OR “Periodontal Index”[Mesh] OR gingival bleeding index OR calculus index OR simplified oral hygiene index OR plaque index
4. 1 AND 2 AND 3

After completing the search in the databases, an additional search was done through the references of selected articles, Google Scholar and by consulting experts.

### 2.3 Selection of the studies

The title, abstract and the full text of the articles were examined according to inclusion criteria until the relevant studies were selected. This process was conducted separately by two researchers. The correlation coefficient between the two researchers was evaluated as 0.94 for the review of the abstracts and 1 for the full text of the articles. Any disagreement between researchers was resolved by the judgment of a third researcher. For each study article author name, publication year, sample size/participant age and sex, decayed, missing and filled teeth in permanent (DMFT index) and primary teeth (dmft index), plaque index, gingival index, bleeding index and DDE were collected. (Table. 1).

**Table 1:** Evidence table of main characteristics of studies included in the systematic review.

Author	Sample size CKD/healthy	Age	DMFT		plaque index		Gingival index		Bleeding index		DDE^b^	
			CKD	Healthy children	CKD	Healthy children	CKD^a^	Healthy children	CKD	Healthy children	CKD	Healthy children
Claudio Andaloro/2018 Italy /	65/61	CKD = 9.92 ± 2.75 Healthy = 9.34 ± 2.43 Overall = 5–16	DMFT = 10.69 ± 1.81 dmft = 12.11 ± 2.03	DMFT = 6.86 ± 1.69 dmft = 7.02 ± 1.75			MGI normal = 11(16.9 %) Very mild = 10 (15.4 %) Mild = 13 (20.0 %) Moderate = 16 (24.6 %) Severe = 15 (23.1 %)	Normal = 20 (32.8 %) Very mild = 14 (23 %) Mild = 11 (18.0 %) Moderate = 11(18.0 %) Severe = 5 (8.2 %)			Hypoplasia = 3.57 ± 0.51	Hypoplasia = 0.09 ± 0.25
O Baygin/2016/ Turkey	17/35	3–18	DMFT = 0.15 ± 0.08 dmft = 0.17 ± 0.13	DMFT = 0.2 ± 0.2 dmft = 0.08 ± 0.11	1.49 ± 0.7	1.59 ± 0.61			1.59 ± 0.61	1.49 ± 0.7	Hypoplasia = 7(41.1 %)	Hypoplasia = 11(31.4 %)
Rubens Caliento2018/ Brazil	25/50	CKD = 9.26 ± 4.01 Healthy = 8.52 ± 2.5	DMFT/dmft 1.92 ± 2.56	DMFT/dmft 1.04 ± 1.42	5.64 ± 11.97	3.44 ± 4.77	CKD = 3.80 ± 6.02	2.64 ± 5.40			Hypoplasia = 10 (40.0 %)	Hypoplasia = 6 (12.0 %)
Davidovich E/2005/ Israel	22/38	Pre-dialysis = 10 ± 0.57 H = 12.6 ± 1.04			1.09 ± 0.08	Pre-dialysis = 1.45 ± 0.11	Pre-dialysis = 1.70 ± 0.12	0.38 ± 0.09	Bleeding on probing = 3.91 ± 0.39	Bleeding on probing = 0.92 ± 0.30	Hypoplasia Pre-dialysis = 3.41 ± 0.34	Hypoplasia = 0.00 ± 0.25
Mateusz Maciejczyk/2019/ Poland	30/30	CKD = 12.93 ± 0.64 Healthy = 12.5 ± 0.8	DMFT = 3.2 ± 0.5 Dmft = 10.1 ± 0.5	DMFT = 3.3 ± 0.5 dmft=11.5 ± 0.4			0 ± 0.2 stage 1 = 0 ± 0.2 stage 2 = 0 ± 01 stage 3 = 0 ± 0.2 stage 4 = 0 ± 0.2 stage 5 = 0 ± 0.2	0 ± 0.2	PBI = 0 ± 0.3 Stage1 = 0 ± 0.1 Stage2 = 0 ± 0.3 Stage3 = 0 ± 0.3 Stage4 = 0 ± 0.3 Stage5 = 0 ± 0.3	0 ± 0.1		
Berkant Sezer/2022/ Turkey	62/52	Stage 1–3 = 9.2 ± 3.4 Stage 4–5 = 11 ± 2.5 Healthy = 9.8 ± 2.6	Median (Q1– Q3) 8.00 (1.00–13.00)	Median (Q1– Q3) Stage 1–3 = 1.00 (1–4) Stage 4–5 = 0.00 (0–2.5)							Stage 1–3 = 66.7 % demarcated opacities = 59.3 % diffuse opacities = 7.4 % Stage4–5 = 80 % demarcated opacities = 62.9 % diffuse opacities = 17.1 %	44.2 % demarcated opacities = 42.3 % diffuse opacities = 1.9 %
Taciana Mara Couto Silva/2019/ Brazil	100/100	CKD = 13.04 ± 2.57 Healthy = 13.04 ± 2.57	Caries free = 31 Low severity = 15 Moderate severity = 26 High severity = 28	Caries free = 67 Low severity = 23 Moderate severity = 10 High severity = 0	Good oral hygiene = 83 Moderated oral hygiene = 11 Poor oral hygiene = 6	Good oral hygiene = 20 Moderated oral hygiene = 18 Poor oral hygiene = 62	Absene of inflammation = 11 Soft inflammation = 12 Moderate inflammation = 23 Severe inflammation = 54	Absene of inflammation = 70 Soft inflammation = 18 Moderate inflammation = 9 Severe inflammation = 3			Without DDE n = 34 Opacity n = 26 Hypoplasia n = 40	Without DDE n = 90 Opacity = 2 Hypoplasia = 8
Julita Szulimowska/2023/ Poland	30/30	9–16	Median DMFT = 10 dmft = 3	Median DMFT = 10 dmft = 3	20 %	23 %	Median GI = 0.2	Median GI = 0.2				

a Chronic Kidney Disease

b Developmental Defect of Enamel

### 2.4 Risk of bias assessment

The quality of the included studies was evaluated by the National Institute of Health (NIH) checklist (https://www.nhlbi.nih.gov/health-topics/study-quality-assessment-tools). According to this checklist, the purpose of the study, the studied population, selection of samples, variables, data collection and measurements, statistical methods and the results of each article were independently assessed by two persons. Any disagreement between researchers in critical appraisal of the studies was resolved by the judgment of third researcher. The checklist for case-control studies consisted of 12 items. Score of 75 %or greater indicated good methodological quality of the article. If an article scored 50 to 75 %, it was of fair quality, and if it scored less than 50 %, the quality of that study was poor.

### 2.5 Statistics analysis

Meta-analysis was conducted to assess the relationship between oral hygiene indices and CKD in children by Comprehensive Meta-Analysis Software Stata 17 (Stata Corp, College Station, TX, USA). Mean and standard deviation values for DMFT, dmft, plaque index, gingival index and the prevalence of DDE in children with CKD and healthy children were extracted for statistical analysis.

To assess heterogeneity in the studies the p-value and I^2^ statistic were utilized. A p-value < 0.05 or an I^2^ > 50 % indicated heterogeneity. A forest plot was employed to illustrate the results of the *meta*-analysis. The Egger's test was used to evaluate publication bias. The significance level was considered at P < 0.05.

## 3 Result

### 3.1 Selection of the articles

**Fig. 1:**
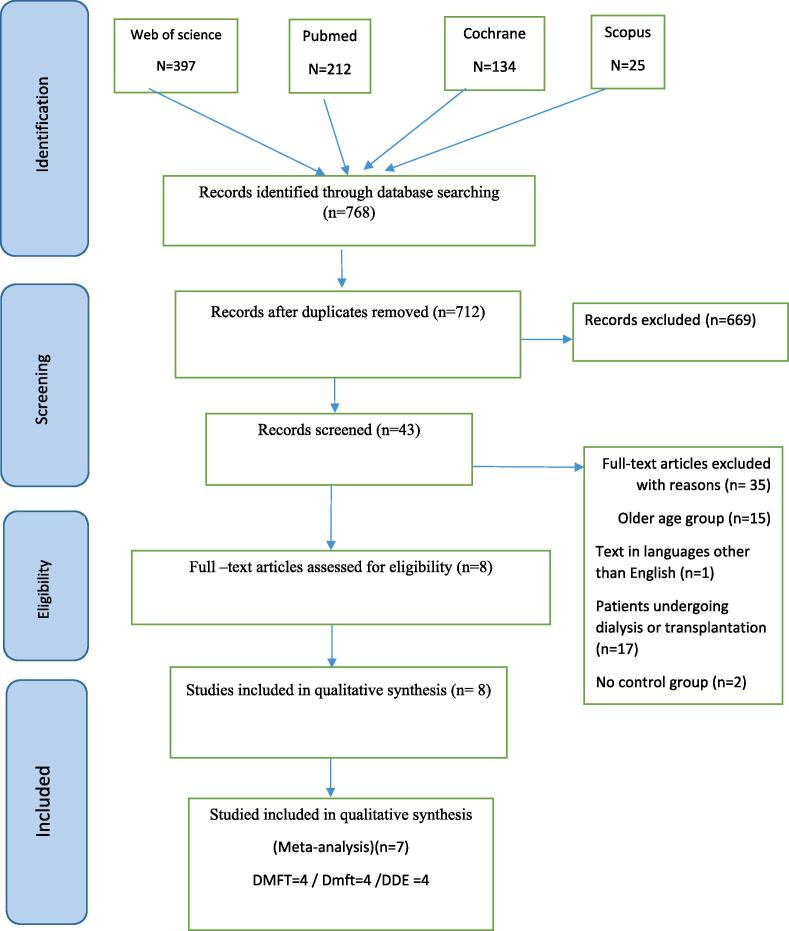
Study flow diagram on the identification of eligible studies.

A total of 768 articles in electronic databases were identified in the search process. 212 articles in the Pubmed, 397 in the Web of Science, 25 articles in the Scopus and 134 articles in the Cochrane databases were found. After removing duplicate articles, 712 articles remained. According to the approach mentioned in article selection, 669 articles were excluded based on the title and abstract and 35 articles based on the full text. Also, to complete the search process, an additional search was done by checking the references of selected articles and consulting with experts in this field, but no new article was added. Finally, 8 articles were selected for quality assessment. (Fig. 1).

### 3.2 Risk of bias

All 8 case control articles received the required score from the NIH quality assessment checklist. Six articles were of high quality and 2 studies were of fair quality.

### 3.3 Study characteristics

The studies were conducted from 2005 to 2023 in different countries including Turkey, Italy, Brazil, Poland, and Israel. The age range of the children varied among the studies, ranging from 3 to 18 years old, with most falling into the late mixed dentition stage. To reduce confounding factors, the caries of primary teeth (dmft) and permanent teeth (DMFT) were examined separately.

CKD has five stages, with the final stage potentially leading to dialysis or kidney transplant. Patients included in the study should not have a history of kidney transplant or dialysis due to the severe dietary restrictions, liquid limitations, and frequent hospital visits associated with dialysis treatment. (Luyckx et al., 2020). Additionally, children with kidney transplants are subject to immune system suppression, making them more susceptible to infections in the oral cavity. (Osiak et al., 2020).

To minimize confounding factors in the advanced stages of the disease, such as various medications and immunosuppression, patients in the advanced stages of transplant and dialysis were excluded from this study. In the later stages of the disease, children may struggle to maintain good oral hygiene, which could negatively impact the results.

In four studies (Andaloro et al., 2018, Baygin, 2017, Maciejczyk et al., 2019, Szulimowska et al., 2023), the caries in two groups of children with CKD and healthy children were compared using the mean of dmft and DMFT indices. In the study conducted by Sezer et al. (Sezer et al., 2023), the median of DMFT was reported. In Caliento et al. (Caliento et al., 2018) study, the caries in primary and permanent dentition were not reported separately and reported as dmft/DMFT that was not comparable with others.

In four studies (Baygin, 2017, Caliento et al., 2018, Sezer et al., 2023, Silva et al., 2019) the prevalence of DDE was compared in CKD and healthy children. For DDE in two studies (Andaloro et al., 2018, Davidovich et al., 2005), unlike other studies included in the *meta*-analysis, the severity of the enamel defect was classified based on the most severe hypoplasia found in any tooth, and it was reported as the mean of severity of the enamel defects, which was not comparable with the prevalence of defects that was investigated in other studies.

Various indices were used in the studies to assess oral health conditions. In the one study (Davidovich et al., 2005), the Lo & Silness plaque index was used (Löe, 1967). In two studies (Baygin, 2017, Caliento et al., 2018), the visible plaque index (VPI) was used. In another study (Szulimowska et al., 2023), Approximate Plaque Index (API) was used to determine the percentage of the tooth surface covered with plaque. In the Silva et al. study (Silva et al., 2019), oral hygiene status was determined by detecting dental biofilm in clinical examination. Due to the use of different indices in the studies, it was not possible to conduct a *meta*-analysis on oral hygiene.

Different indices were used in the studies to compare gingivitis in CKD and healthy children. In two studies (Davidovich et al., 2005, Maciejczyk et al., 2019), the mean of the gingival index was reported, but in the study of Szulimowska et al. (Szulimowska et al., 2023), the prevalence of different stages of gingival index was reported. In Silva et al. (Silva et al., 2019) study, the median of gingival index was measured. Two other studies (Andaloro et al., 2018, Caliento et al., 2018) also used the modified gingival index (MGI), which is a non-invasive (probe-free) method to evaluate the gingival condition.

The bleeding index was also evaluated to assess gingivitis. In two studies (Baygin, 2017, Davidovich et al., 2005), the mean of number of places that bleed during probing was reported, and in Maciejczyk et al. study (Maciejczyk et al., 2019), the intensity of bleeding from the gingival sulcus (SBI) after probing was reported. Therefore, it was not possible to conduct a *meta*-analysis due to the inconsistency of the reports.

### 3.4 Quantitative analysis

#### 3.4.1 The relation between CKD and dental caries (DMFT and dmft)

Four studies (Andaloro et al., 2018, Baygin, 2017, Maciejczyk et al., 2019, Szulimowska et al., 2023) with similar methods compare the mean of DMFT and dmft in CKD and healthy children. The heterogeneity was observed among these four studies in DMFT (I^2^ = 95 %, p-value < 0.001) and dmft (I^2^ = 98.2 %, p-value < 0.001). Hence a random-effects model was used for *meta*-analysis. The results found no statistically significant difference in mean of DMFT and dmft scores in CKD and healthy children with a mean difference of −0.433 (95 % CI: −1.689 to 0.823; p-value = 0.500) and-0.095 (95 % CI: −2.240 to 2.051; p-value = 0.931) respectively. (Table. 2 and Table 3) Based on the Beggs and Mazumdar test, no publication bias was observed for DMFT (P = 0.089) and dmft (P = 0.30), so the trim and fill method was not used.

**Table 2:** Forest plot of DMFT in children with chronic kidney disease and healthy children.

**Table 3:** Forest plot of dmft in children with chronic kidney disease and healthy children.

#### 3.4.2 The relation between CKD and DDE

Four studies (Baygin, 2017, Caliento et al., 2018, Sezer et al., 2023, Silva et al., 2019) with similar methods compare the prevalence of DDE in CKD and healthy children. The heterogeneity was observed among these four studies (I^2^ = 78.5 %, p-value = 0.003). Hence a random-effects model was used for *meta*-analysis. CKD had a significant effect on the DDE index in children with an effect size of 4.916 (95 % CI: 1.752 to 13.799; p-value = 0.002). Therefore, having CKD can increase the chance of DDE in children by 4.9 times. (Table.4) Based on the Beggs and Mazumdar test, no publication bias was observed (P = 0.308), so the trim and fill method was not used.

**Table 4:** Forest plot of DDE in children with chronic kidney disease and healthy children.

## 4 Discussion

The prevalence of CKD in most countries is more than 10 % (Eckardt et al., 2013). Patients with CKD are highly susceptible to various infections due to the overall weakening of the body, reduced immunological response, and the masking of infection signs and symptoms by medications. CKD can lead to systemic changes and oral complications, affecting the teeth, oral mucosa, bone, periodontium, salivary glands, salivary flow and composition, tongue, oral cavity, and temporo-mandibular joint (Luyckx et al., 2020, Oyetola et al., 2015).

Decreased salivary flow in CKD patients increases the likelihood of symptoms such as xerostomia, dysphagia, changes in taste, burning sensation in the mouth, as well as oral diseases like stomatitis, cavities, periodontal diseases, urea smell, pale mucous membranes, enamel defects, and candidiasis infections. These issues can significantly impact the quality of life in children with CKD (Karthik and Gheena, 2021, Amrollahi et al., 2023) Although children represent only a small population of all patients with CKD, they have special challenges because of the many extra renal manifestations of CKD that complicate the management. (Sezer et al., 2023).This systematic review was conducted to assess the relationship between CKD and oral and dental health indicators in children.

The systematic review results revealed that CKD can increase the incidence of DDE by 4.9 times. However, it does not have any effect on the dental caries in primary and permanent teeth. In a qualitative analysis, children with CKD who maintain regular oral hygiene instructions, seems to have good periodontal health.

Out of the 10 studies analyzed in the systematic review, 7 were also summarized using *meta*-analysis. Four studies were reviewed for DMFT/dmft (Andaloro et al., 2018, Baygin, 2017, Maciejczyk et al., 2019, Szulimowska et al., 2023) In these four studies, all participants were examined according to the World Health Organization criteria. (WHO) and the scores were reported as mean of DMFT and dmft. Additionally, the caries index was reported for permanent and primary teeth separately. In the one studies (Caliento et al., 2018), DMFT and dmft indices were not investigated and reported separately. In another study (Sezer et al., 2023) the median DMFT was reported. In the study by Silva et al.(Silva et al., 2019), dmft and DMFT indices were reported qualitatively and scored as low, medium or high intensity, with the number of people in each group reported.

Four studies for DDE (Baygin, 2017, Caliento et al., 2018, Sezer et al., 2023, Silva et al., 2019) were reviewed. Their reports expressed the number of people in whom DDE were observed. In two studies (Andaloro et al., 2018, Davidovich et al., 2005), unlike other studies included in the *meta*-analysis, the severity of the enamel defect was classified based on the most severe hypoplasia found in each tooth.

Various indices were used in the studies to check the oral health condition. As a result the Meta analysis was not possible for this variable. In four studies (Davidovich et al., 2005, Baygin, 2017, Caliento et al., 2018, Szulimowska et al., 2023) no differences were observed in oral health condition between the two groups with CKD and the healthy group. However, in one study (Silva et al., 2019), the number of individuals with poor hygiene among children with CKD was significantly higher, potentially due to the use of a different index.

Different indices were used in the studies to evaluate gingivitis in children with CKD making it impossible to conduct a *meta*-analysis on this variable. In three studies (Caliento et al., 2018, Maciejczyk et al., 2019, Szulimowska et al.), no significant difference was observed in the gingival condition between CKD patients and the control group. However the results of three studies (Davidovich et al., 2005, Andaloro et al., 2018, Silva et al., 2019) indicated a higher percentage of severe gingivitis in children with CKD. The variation in results may be related to differences in sample size and indices used for evaluating gingivitis.

In Silva et al. (Silva et al., 2019) study, a significant difference in gingival index was noted due to the presence of more biofilm in these patients, suggesting that poor oral hygiene can contribute to gingivitis. Additionally, the use of different index to assess oral hygiene in this study may influenced the results obtained.

The bleeding index was also considered to evaluate gingivitis in children with CKD. In two studies (Baygin, 2017, Maciejczyk et al., 2019), no differences were observed between children with CKD and healthy children. However, in the study by Davidovich et al. (Davidovich et al., 2005), children with CKD had more bleeding in the gingiva. In assessment of gingival health in children with CKD, most studies have shown that if there is minimal plaque accumulation on the tooth surfaces and good oral health, the gingival health remains desirable regardless of the presence of CKD.

The *meta*-analysis revealed a significant relationship between CKD and DDE, which may be attributed to dysfunction of ameloblasts. Factors such as hypocalcemia, decreased serum levels of hydroxycholecalciferol (active form of vitamin D), increased serum levels of phosphate and parathyroid hormone can disrupt ameloblast activity. (Subramaniam et al., 2012, Salanitri and Seow, 2013).

There was no significant relationship between dental caries (DMFT and dmft) and CKD. Previous studies had reported conflicting results in this field. Despite the presence of a large amount of urea in saliva and an alkaline pH, we anticipated lower prevalence of dental caries in these patients. (Scannapieco and Cantos, 2016, D'Souza et al., 2023).

On the other hand decreased saliva and dry mouth make these patients more susceptible to caries. Dry mouth is a multi-faceted phenomenon, one cause of which is reduced salivary flow and limited water consumption in these patients (Nylund et al., 2018). Consequently, the interaction of various factors in patients with CKD causes no significant difference in prevalence of dental caries in primary and permanent teeth when compared with healthy children. One of the limitation of this study was the use of different indices to report the results which made it difficult to summarize findings, particularly in the field of gingival health and oral hygiene.

## 5 Conclusion

The presence of CKD can increase the incidence of DDE by 4.9 times, but it has no significant effect on the prevalence of dental caries in primary and permanent teeth. In a qualitative summary, it appears that gingival health in children with CKD is good if they maintain good oral hygiene. However more studies are needed in this field to provide more definitive result.

## Declaration of competing interest

The authors declare that they have no competing financial interests or personal relationships that could have appeared to influence the work reported in this paper.
